# Castration of Cryptorchids1Read at a meeting of Alabama Veterinary Association.

**Published:** 1895-12

**Authors:** G. W. Browning

**Affiliations:** Huntsville, Ala.


### CASTRATION OF CRYPTORCHIDS.[1]

By G. W. BROWNING, V.S.,
HUNTSVILLE, ALA.

Cryptorchidy in horses as an abnormal condition becomes a serious matter by reason of the great depreciation of money-value of the animals which are affected with it. It is accompanied with an ugly disposition, which at times renders their temper uncontrollable and becomes the cause of great difficulties and serious dangers for those who have them in charge. Excited by the presence of other animals of their own species, either male or female, the ridgling becomes nervous and noisy, rears and kicks, resists control, makes violent attacks on other animals, and exposes himself by his excitement to numerous accidents. Prevented from satisfying his sexual desire, he often vents his rage upon his driver with foot and tooth in a most treacherous manner. At times it is sufficient for his keeper to have merely touched other horses or mares before approaching him to excite him suddenly into a copulative rage. He snorts fiercely, jerking himself and whinnying with the peculiar and the characteristic tone of the animal under sexual desire, bends his neck convulsively, strikes out with his forefeet, and often, unless held firmly and shortly in hand,

[1] Read at a meeting of Alabama Veterinary Association.

rushes furiously upon his keeper. It is true that sometimes he can be kept under control by excessive work and low diet, but he is always to be held under suspicion, and his intermittent and sudden outbreaks of aggressive temper are always to be feared after the enjoyment of a few days' rest or liberal feeding. Hence, their comparatively low value and the difficulty met with in disposing of them by sale, and the great danger to their keepers and to surrounding animals which are generally of more value than they, make it necessary for veterinarians to undertake the operation for the missing testicle.

Should we be called upon to castrate a ridgling we should operate in such a manner as to be in the least danger of destroying our patient and to be of the most credit to ourselves as operators. Should we follow the greater amount of literature on the subject this would be hard to do; for there is so much confusion and difference of opinion among writers on this subject that it is hard for one who reads them to decide which is the best method until he has considerable experience of his own. I desire to state that the opinions herein expressed are the results of my individual experience and are free from envy, hatred, and all uncharitableness.

Now the question comes in, On what cases is it practical to operate? My answer to this is, When you have a ridgling two years of age and in a healthy condition. I would not advise any one to operate on a ridgling under this age, or one not in a healthy condition.

ANATOMY. The first condition of success in the castration of cryptorchids is a perfect knowledge of the direction to follow in the inguinal region in order to reach the testicle in ectopia. The most perfect asepsia would be powerless to insure success if the hand of the operator should follow a wrong road during this very delicate step of the operation; and for this reason I have deemed it indispensable to say a few words as to the topographical anatomy of the region before entering upon a description of the *modus operandi*, in order better to familiarize one with the operation among veterinarians. It will be of advantage to give you a description, as plain and accurate as possible, of the essential organs and their most important relations. The inguinal region will not be described as given by anatomists, and I shall only indicate the parts of which a knowledge is indispensable, first considering the inguinal region itself and subsequently the testicles and the various positions they may occupy.

*Inguinal region.* This is constituted by the angle formed by the union of the inferior abdominal wall with the internal crural region, running obliquely downward, inward and from before backward. It extends from the external angle of the ilium to the anterior border of the pubes, offering for consideration the inguinal ring and the inguinal tract, or interstice, of which a description possesses special interest in considering this operation.

We will first glance at some of the anatomical dispositions presented by the fleshy portion of the small oblique muscle of the abdomen and the crural arch, which are the anterior and posterior boundaries of the entrance for the initial procedure of the operator. The fleshy portion of the small oblique triangular and flabelliform proceeds from the external angle of the ilium and the superior and external quarter of the crural arch; its posterior border, slightly elevated, simply rests upon the crural arch to which it is united by loose connective-tissue, except on a level with the inguinal ring where the two organs are separated; at this point the small oblique is covered by the aponeurosis of the great oblique, which, very thin and almost connective-tissue, twists by its archiform fibres around the posterior, or external, lip of the inguinal ring. The crural arch is a wide fibrous band, very strong and resisting, attached by one extremity to the external angle of the ilium, and by the other to the anterior border of the pubes; spreads itself upon the pelvi-crural muscles, and, diminishing in structure, enters upward into the abdominal cavity. In the superior quarter of its length it assists in the attachment of the small oblique muscle, and in the rest of its extent it simply rests upon the posterior border of this muscle, thus coöperating in the formation of the inguinal canal and of the inguinal ring.

*The external or inguinal ring.* This is an oval opening easily felt under the skin near the anterior border of the pubes at the point of union of this border with the prepubic tendon of the abdominal muscles. Its greater diameter is obliquely directed from before backward and from without inward. Piercing through the aponeurosis of the great oblique of the abdomen, the inguinal ring, exposed by the incision of the scrotum and of the dartos, with the laceration of the connective-tissue underneath, presents two lips or borders—one anterior or internal, the other posterior or external—and two commissures, an anterior and a posterior. The anterior, or internal, border is formed by the posterior border of the small oblique; the posterior, or external, border is formed by the crural arch. The internal, or peritoneal, ring is merely a dilated slit between the small oblique muscle and Poupart's ligament.

The inguinal tract or interstice (because in cryptorchids there is as yet no inguinal canal) is situated between the posterior border of the small oblique of the abdomen and the crural arch. It runs obliquely from above downward, from before backward, and from without inward; and is about two and one-half to three inches long.

CASTING. Having briefly run over the anatomy of the parts under consideration, it will not be amiss to say something about securing the animal for operation. I cast by the old Rarey method, modified to suit my convenience. I like this way, because it lets a horse down easily. The horse should always be cast on the side opposite the ectopia, and so fastened by crossing the ropes on the back and flexing the leg by bringing the fetlock-joint up against the stifle, which insures the proper exposure of the inguinal region to the best advantage for the operator. I always operate with the horse lying on his side, unless it be a double cryptorchid, when I turn him on his back. Some operators recommend not to operate on a double cryptorchid at one sitting, but operate on one side, and when it gets well operate on the other. I think that sounds too much like the man amputating his dog's tail, taking off an inch at a time to keep it from hurting so bad.

OPERATION. The animal being secured, the inguinal region and surrounding parts should be thoroughly washed with soap, well wiped out, and then submitted to a second washing with a bichloride of mercury solution. The hands of the operator and his assistants should be washed with the same solution. The instruments, consisting of a convex bistoury, écraseur, and torsion forceps, should be put in a vessel containing a creolin solution.

*Modus operandi.* Take up a transverse fold of the scrotum with the left hand and pull well forward, putting it on the stretch; with a convex bistoury make a longitudinal incision parallel with the raphe, cutting through the scrotum and dartos, being careful not to sever any of the bloodvessels which abound in this region. Then with the fingers divide the connective-tissue; dip the hand in a mixture of oil and creolin, consisting of one ounce of creolin to twelve ounces of olive oil; using the left hand if the testicular ectopia is on the left side, the right hand if on the right side. With the fingers united in the shape of a cone the hand enters the external ring (which is readily felt), keeping the back of the hand to the small oblique muscle and the pulp of the fingers on the anterior face of Poupart's ligament, passing up the inguinal interstice in a direction toward the external angle of the ilium. On reaching the internal, or peritoneal, ring break through the peritoneum at its upper border an opening large enough to pass one or two fingers. At this point the operator should be very careful, and use his head as well as his hands; go slowly, and do not get excited. If the horse has been properly tied and the inguinal region so exposed as not to cramp the arm of the operator, he will have little trouble in securing the testicle, epididymis, or the suspensory ligament. It is not necessary, one time in a hundred, to introduce the whole hand into the abdominal cavity. If the testicle cannot be readily felt, then turn the fingers backward in a circular motion and feel for the suspensory ligament of the testicle (which is a double fold of peritoneum extending from the sublumbar region to the anterior border of the pubes), and by using gentle traction on this the testicle can be drawn to the opening and removed. The testicle varies in size from a hazelnut to a hen's egg, and is generally flabby and not very well developed. We sometimes find a large, abnormal globus minor situated in the inguinal canal and the testicle remaining in the abdominal cavity, which is apt to mislead the inexperienced operator, thinking he has removed the true testicle. I recollect once operating on a case of this kind, and found what I took for the testicle lodged in the inguinal canal; but on a closer examination I found it to be a large globus minor, and by using a little force brought out the true testicle, which was fully five inches distant from the globus minor. In removing the gland I always use the écraseur, considering it the easiest of application, safest, and quickest.

After the removal of the testicle, I wash out the wound thoroughly with creolin, one ounce, and water, one quart; then pour in a little of the creolin and oil, and let the horse rise; have him walked slowly for an hour to prevent him from lying down from any colicky pains he might have; then I have him tied up high for twenty-four hours. After this, I order him walked a little every day, and use hot fomentations to which a little creolin has been added, and keep the wound open by the finger dipped in creolin and oil.

*Accidents.* The three principal complications which may be met with after the castration of cryptorchids are hemorrhage, eventration, and peritonitis. As for hemorrhage, I never had one to bleed enough to prove fatal, and I have had but two cases of eventration, and one of those was a ridgling that a quack had undertaken to operate on about two hours before I was called, and had failed to find the testicle, and had left the wound in a horribly mangled condition. When I went in and brought out the testicle, I had an escape of about three feet of small intestine, but succeeded in returning it, and took a deep circular suture. Except a very large swelling, the horse did very well. The other case was caused by the owner not carrying out my directions, allowing the horse to lie down and roll with colicky pains. The horse trampled on the intestine and lacerated it in such a manner as to have to be destroyed. I have had more trouble with peritonitis than anything else. It generally comes on from three to five days after the operation. I recollect having but one case of tetanus.

*Prognosis.* If the horse is in a good, healthy condition, and the operator has taken proper care in operating by using cleanliness with thorough antiseptics, the results are generally successful and rarely fatal. In a practice of fifteen years I have operated on from thirty to eighty cryptorchids each year, with a loss of about four per cent. This includes a number of cases in which the cryptorchid had becn operated on by other parties who had failed to find the testicle. As a rule, an operation of this kind will not prove as successful as one that has never been tampered with; for when the animal has once been operated upon without success, the healing of the wound produces a deep, thick cicatrix, which is almost impossible to go through in passing up the inguinal region. In subjects of this kind I make my incision in the scrotum a little in front of this cicatrix, and keep in front all the way till I puncture into the abdominal cavity, for I have found it most always the case where one failed to castrate a cryptorchid that the operator went too far back, passing behind Poupart's ligament, and therefore missing the testicle entirely.

I never use anæsthetics. I consider the operation not sufficiently painful to indicate their use, and they are objectionable because in destroying sensibility there is no contraction of the suspensory ligament when the hand of the operator takes hold of it or the testicle. Therefore, for this reason, I consider anæsthetics objectionable and calculated to mislead the operator.

I will now mention what I consider some of the causes of failure to castrate in this operation. First, the operator strikes too difficult a subject before he has had sufficient experience. Second, by not tying the horse properly and spreading the legs away from the inguinal region and producing pressure on the hand of the operator and destroying the sense of touch. Third, by the operator going too far back, passing the hand up behind Poupart's ligament, and instead of puncturing the peritoneum at the proper place pushes the peritoneum off from the sublumbar muscles, thus placing an obstacle between the hand of the operator and the testicle. Hence, the importance of taking the right course when first entering the external inguinal ring.

I should like to give you the history of some of the cases of operations on cryptorchids I have encountered, but this paper is already longer than I expected it would be.

